# Nursing Students’ Acceptance Intention of a Smart Device, Information Literacy, and Problem-Solving Confidence

**DOI:** 10.3390/healthcare9091157

**Published:** 2021-09-03

**Authors:** Eun-Jin Choi, Jeong-Hye Park, Se-Won Kang

**Affiliations:** 1 Department of Nursing, Ulsan College, Ulsan 44022, Korea; ejchoi@uc.ac.kr; 2 Department of Nursing, Gyeongsang National University, Jinju 52725, Korea; masternur@gnu.ac.kr; 3 Department of Nursing, Dongseo University, Busan 47011, Korea

**Keywords:** acceptance intention, information literacy, problem solving

## Abstract

The purpose of this study was to investigate the relationship between nursing students’ acceptance intention of a smart device, information literacy, and problem-solving confidence to explore the factors that may improve nursing students’ problem-solving confidence. Data were collected from 450 nursing students between July and August 2019 in two cities in Korea. The results showed that there is a positive correlation between problem-solving confidence and acceptance intention (r = 0.353, *p* < 0.001) and between problem-solving confidence and information literacy (r = 0.501, *p* < 0.001). Further, participants with high acceptance intention of a smart device and information literacy scores had significantly higher problem-solving confidence (t = 5.447, *p* < 0.001; t = 10.072, *p* < 0.001) than participants with low acceptance intention. In addition, in the logistic regression the odds ratio between the acceptance intention of a smart device, information literacy, and problem-solving confidence groups was odds ratio 2.071 (*p* < 0.001, CI: 1.412–3.037) and odd ratio 4.966 (*p* < 0.001, CI: 3.298–7.477). To improve nursing students’ problem-solving confidence, educational strategies should be developed to build information experience and information utilization capabilities.

## 1. Introduction

The fourth industrial revolution is the main focus of recent academic and industrial fields. The fourth industrial revolution changes the boundaries between science and technology, such as artificial intelligence (AI), robots, the Cloud, and the Internet of Things. Therefore, the boundaries between reality and virtual reality and between machines and humans are blurred [1]. It has the potential to radically change social systems, including the medical field, and the manner in which healthcare is provided and implemented [2,3]. Changes in this industry can change the structure and function of the healthcare system itself or affect all the processes of interaction between patients and healthcare practitioners [4]. In particular, it is likely that cooperative forms of technology that link with humans will be developed [5]. In the future, environmental changes in society are expected to change the work of nurses and the extent of problem solving required [6].

In addition, as the proliferation of smart devices equipped with AI is rapidly spreading, the interest in smart devices is increasing [7,8], and consumer acceptance of these smart devices has had a huge impact on consumer life. Smart devices are collectively known as various terminal devices and their controllers, such as sound equipment, healthcare, imaging equipment, driving devices, such as drones, radio frequency cars, helicopters, etc. smart devices also use various communication technologies such as Wi-Fi, Bluetooth, Long-Term Evolution, etc., as well as being designed as wearable devices, smart home appliances, augmented reality devices, such as virtual reality/mixed reality, and various other types of products [9].

The acceptance intention of a smart device involves the intention, plan, and recommendation to use the smart device presently or in the future [10]. There is a theory describing the acceptance process of the smartphone device, which uses the Technology Access Model. This is based on the theory that the user’s attitude toward the use of information technology determines the intent of the act and the behavior in their use of information technology, which is determined by the intent of the act [11,12,13]. In healthcare, the acceptance intention of a smart device was conducted in some influencer studies [14,15,16].

Information literacy is the ability to recognize the need for information, which is related to knowledge and technology, and to efficiently collect, analyze, interpret, and evaluate information. As informationization is accelerated in nursing organizations, work processes and performance creation are required, and information literacy is deemed important [17,18]. Information literacy is related to the problem-solving abilities of nurses [19,20]. In addition, the results of a study on nursing students demonstrated that information literacy affects evidence-based performance skills [21,22,23]. 

Problem-solving confidence refers to the degree of confidence in one’s ability to solve problems [24]. It is defined as a belief in one’s ability to address problems effectively and to have a sense of self-efficacy in the aspects of problem solving [25]. High self-confidence in problem solving means that when an individual solves a problem, they address all the tasks involved in the problem. It is significantly related to students’ positive learning outcomes and their work performance [26]. Kim’s study of college students indicated that problem-solving confidence has a positive effect on career compliance [27]. Confidence in their ability to solve problems efficiently can determine whether an individual can respond successfully when faced with a problem situation [28]. Ultimately, it is necessary to develop these competencies by identifying factors, which can increase confidence in problem-solving among nursing students in the undergraduate program.

In addition, many hospitals already utilize a variety of information technology (IT) devices for patient care and nursing, and the introduction of these systems is expected to expand further in the future. Therefore, in the fourth industrial era, it will be necessary to increase nursing students’ ability to respond well to changes and their problem-solving confidence. In this study, we sought to determine its relevance by selecting the acceptance intention of a smart device and information literacy as a factor that can improve problem-solving confidence.

**Figure 1:**
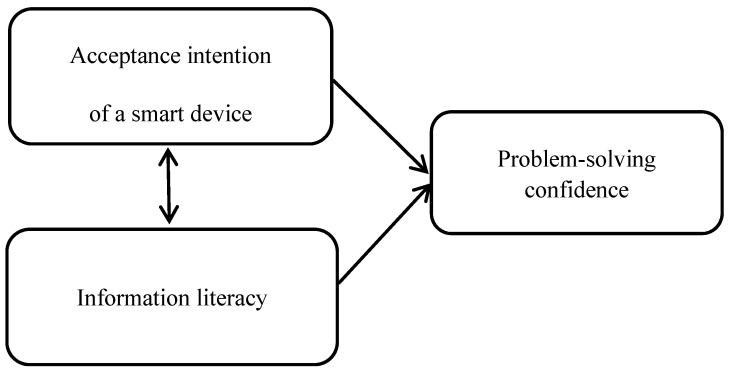
The hypothesized model.

This study aimed to investigate the relationship between the acceptance intention of a smart device, information literacy, and problem-solving confidence, as well as to explore factors that may improve the problem-solving confidence of nursing students (Figure 1). 

We aimed to:

- Identify the acceptance intention of a smart device, information literacy, and problem-solving confidence;
- Identify the relevance of the acceptance intention of a smart device, information literacy, and problem-solving confidence; and
- Identify the differences in problem-solving confidence according to the acceptance intention of a smart device and information literacy.

## 2. Materials and Methods

### 2.1. Study Design

This study was designed as a descriptive research study to investigate the relationship between the three variables and to identify the variables affecting problem-solving confidence.

### 2.2. Participants and Data Collection

The participants were third and fourth year nursing students who had experience in clinical practice at universities in U and P cities in Korea. Participants were selected by using convenience sampling. There were 450 participants in the study: 394 female students (87.6%) and 56 (12.4%) male students. There were 194 (43.1%) third year students and 256 (56.9%) fourth year students. The average age was 21.6 years. 

Data were collected from July 2019 to August 2019. The survey consisted of a self-report questionnaire. It took approximately 10 to 15 min to complete. 

The number of study participants was the minimum sample size, which was 235 participants, when calculating the sample size to 0.15 with a sample size of 0.05 significance level and 80% power and F-test use using the sample size calculation program G*Power 3 program according to Cohen’s sample extraction formula sufficient for 450 people. 

### 2.3. Ethical Considerations

This study was conducted in accordance with the research procedures of the World Medical Association Declaration of Helsinki (2013) [29], and the Institutional Review Board of Dongseo University for ethical protection of the subjects. Data were collected by using the principle of voluntary participation in the study and for those who provided written informed consent. 

### 2.4. Measurements

#### 2.4.1. Acceptance Intention of a Smart Device

The acceptance intention of a smart device was measured using a tool developed by Jo and Jun [30]. It consists of 26 questions. There are five items of audience innovativeness, three items of self-efficacy, three items of intention to use, three items of perceived usefulness, four items of perceived ease of use, two items of subjective norms, two items of perceived cost, and four items of enjoyment. It is marked from one point, “not at all” to seven points, “very much” on a 7-point Likert scale. The higher the score, the higher the acceptance intention of a smart device. In this study, the reliability of the tool was Cronbach’s α = 0.927., Cronbach’s α is a measure of the internal consistency of a test or scale, expressed as a number between 0 and 1. If the value is 0.7 or more, it is considered appropriate [31].

#### 2.4.2. Information Literacy

Information literacy was measured using a tool originally developed by Straggers et al. [32] and modified by Kim [18] for nursing students. Information literacy consists of 13 items, including six items for information management, five items for informationization recognition, and two items for information search. The score is on a 5-point Likert scale ranging from one which signifies “not at all” to five which signifies “very much”. The higher the score, the higher the level of information literacy. In the present study, the reliability of the tool was Cronbach’s α = 0.921.

#### 2.4.3. Problem-Solving Confidence

Problem-solving confidence is defined as the personal belief and self-confidence in one’s ability to manage problems efficiently [24]. Measurement of problem-solving confidence was performed using a tool developed by Heppner and Peterson [24]. It consists of a total of 11 items, with a 5-point Likert scale ranging from one which signifies “not at all” to 5 which signifies “very much”. The higher the score, the higher the problem-solving confidence. In the present study, the reliability of the tool was Cronbach’s α = 0.853.

### 2.5. Data Analysis

The collected data were analyzed using PASW Statistics 18.0 (IBM, Chicago, IL, USA). The scores of the participants’ acceptance intention of a smart device, information literacy, and problem-solving confidence were analyzed as averages and percentages. The relationship among the three variables was analyzed by using a Pearson’s correlation. To determine the difference in problem-solving confidence according to the acceptance intention of a smart device and information literacy, the acceptance intention of a smart device and information literacy scores were divided into two groups (50% high score group, 50% low score group). The difference in problem-solving confidence scores between the two groups (50% high score group, 50% low score group) was analyzed by using an independent samples *t*-test. To examine detailed differences, the problem-solving confidence score was also classified into two groups (50% high score group, 50% low score group) and the odds ratio between groups in problem-solving confidence, the acceptance intention of a smart device, and the information literacy group was analyzed by using a binary logistic regression. In the logistic regression, the adjusted odds ratio was obtained by controlling for gender and grade. The significance level of the collected data was 0.05. 

## 3. Results

### 3.1. The Degree of the Acceptance Intention of a Smart Device, Information Literacy, and Problem-Solving Confidence

The degree of the acceptance intention of a smart device for participants, information literacy, and problem-solving confidence is demonstrated in Table 1. The score of acceptance intention of a smart device was 4.78 out of 7, the information literacy score was 3.87 out of 5, and problem-solving confidence was 3.40 out of 5.

For information literacy, there was a significant difference by grade (t = −1.975, *p* = 0.048). In addition, for problem-solving confidence, there was a significant difference by gender (t = 3.598, *p* < 0.001).

### 3.2. The Relevance of Acceptance Intention of a Smart Device, Information Literacy, and Problem-Solving Confidence

The relationship between the acceptance intention of a smart device and problem-solving confidence demonstrated a moderate positive correlation (r = 0.353, *p* < 0.001). The relationship between information literacy and problem-solving confidence indicated a positive correlation (r = 0.501, *p* < 0.001). The relationship between acceptance intention of a smart device and information literacy was positively correlated (r = 0.541, *p* < 0.001) (Table 2).

### 3.3. The Difference of Problem-Solving Confidence According to Groups in the Acceptance Intention of a Smart Device, Information Literacy, and Problem-Solving Confidence

In order to observe the difference in the problem-solving confidence according to acceptance intention of a smart device and information literacy, the score of the acceptance intention of a smart device and information literacy was divided into the top 50% (high score group) and the lower 50% (low score group). There were statistically significant differences in problem-solving confidence between the groups. The group with a high acceptance intention of a smart device had a statistically significantly higher score in problem-solving confidence than the low score group (t = 5.447, *p* < 0.001). In addition, the group with a high information literacy score had a statistically significantly higher score in problem-solving confidence than the low score group (t = 10.072, *p* < 0.001) (Table 3). 

In addition, to verify the difference in problem-solving confidence according to the acceptance intention of a smart device and information literacy, the odds ratio (OR) among the groups was analyzed. Gender and grade, which showed differences in the general characteristics’ analysis, were controlled. 

As a result, the logistic regression indicated that the odds ratio between groups in the acceptance intention of a smart device and problem-solving confidence was determined in OR 2.071 (*p* < 0.001, CI: 1.412–3.037). In addition, information literacy and problem-solving confidence were established in OR 4.966 (*p* < 0.001, CI: 3.298–7.477). Therefore, the group with a high score group in the acceptance intention of a smart device, information literacy, and the probability of increasing the score of problem-solving confidence was 2.071 times, 4.966 times greater than that of the low score group, respectively (Table 4).

## 4. Discussion

This study explored the factors that affect problem-solving confidence in nursing students. By grasping the relationship between the acceptance intention of a smart device, information literacy and problem-solving confidence, we aimed to devise a way to increase problem-solving confidence. 

### 4.1. The Acceptance Intention of a Smart Device, Information Literacy, and Problem-Solving Confidence of Participants

The degree score of the participants’ acceptance intention of a smart device was more than halfway at 4.78 points (out of 7). In a study by Park [33], which was measured using the same tool, the degree of acceptance of smartphone healthcare applications was higher than that demonstrated in 3.28 out of 5 in the results of 250 adults aged 20–70 years. As there is a difference in the age distribution of the subjects, it is difficult to compare them according to the study results, and further analysis by age is recommended.

The participants’ information literacy score was 3.87 points out of 5. This result is higher than the 3.44 scored by the hospital nurses in Seo et al. [34] using the same tool. Nursing students who are studying rather than nurses working in the practice field often have a high demand for information literacy, and coding and software-focused education are often compulsory in school education. Therefore, students’ self-reported information literacy may have been higher. In addition, the information literacy score was higher in fourth grade students than in third grade students. Although the school period and clinical practice period according to the grade may affect information literacy, it could be affected by various other factors. Further analysis on the characteristics of information literacy ability is needed.

The participants’ degree of problem-solving confidence was 3.40 points out of 5, which is a score lower than the 3.55 points reported in the study by Han [28] for only fourth year nursing students. Among nursing students, problem-solving confidence is thought to vary depending on their clinical practice experience. During the clinical practice period, students encounter various nursing problems but acquire problem-solving skills through training on such courses. Therefore, the difference in the clinical practice experience of students may affect their problem-solving confidence. It is necessary to examine the differences in problem-solving confidence scores according to the period of their clinical practice experience. Moreover, males scored higher in problem solving confidence than females. These results are similar to those in Han and Yang’s (2021) study [35] targeting nursing students who used the same tool, which showed academic performance, critical thinking, major selection motivation, and gender as influencing factors of problem-solving confidence.

### 4.2. The Relationship among the Acceptance Intention of a Smart Device, Information Literacy, and Problem-Solving Confidence

Among nursing students, the acceptance intention of a smart device and problem-solving confidence of nursing students demonstrated a positive correlation (r = 0.353, *p* < 0.001). This result is similar to the study on the acceptance intention of the healthcare application for 220 adults [36], which indicated that people with high self-efficacy have a high acceptance intention of healthcare applications. Problem-solving confidence illustrates the relevance of acceptance intention of a smart device since it provides a sense of self-efficacy in problem solving.

The relevance of information literacy and problem-solving confidence demonstrated a strong correlation (r = 0.501, *p* < 0.001). This is similar to the results of significantly increased problem-solving ability and evidence-based nursing ability when providing the nursing information capacity enhancement program to 72 nursing students in the study by Ha et al. [19]. In addition, a study of clinical nurses [22] depicts a similar result, as shown in our results, that information literacy has a positive effect on problem-solving ability.

### 4.3. The Differences in Problem-Solving Confidence According to the Acceptance Intention of a Smart Device and Information Literacy

The group with a high score on the acceptance intention of a smart device had a significantly higher problem-solving confidence score than the low score group (*p* < 0.001). The probability of increasing problem-solving confidence was 2.071 times. This is similar to a study [37] that demonstrated that the acceptance of mobile healthcare services may affect self-efficacy. Therefore, the understanding of new technology use and self-efficacy has a positive proportional relationship, which may affect the use and spread of new technology. In other words, becoming acquainted with and familiarizing nurses and nurses in training with various relevant information technologies could increase problem-solving confidence. 

In addition, the group with a high score for information literacy had a significantly higher score on problem-solving confidence than the low score group (*p* < 0.001). The probability of increasing problem-solving confidence was 4.966 times. These results are similar to the study results by Jo and Gu [17], from 208 senior nursing students, which showed a significant direct effect on problem-solving ability and self-directed learning ability. Therefore, information literacy could improve students’ problem-solving confidence. 

The hospital already implements a variety of information and communications technologies (ICT). These include: An electronic medical records system, a dosing management system using barcode/radio frequency identification, nursing performance using a personal digital assistant (vitality signs, blood glucose measurement, etc.), an automatic recording training system, patient education using an iPad, and a quarantine transfer machine. Using various ICTs in all areas of nursing, patient safety is ensured and nursing efficiency is increased. ICT-use in healthcare in the future will continue to expand owing to these advantages. The information literacy required by nurses in a rapidly changing digital healthcare environment will be further emphasized, and information literacy will be linked to nurses’ work performance and problem-solving capabilities.

Therefore, nursing students are required to take the initiative and access and utilize advanced information devices to improve their information utilization ability. Experience in using various smart devices will increase their ability to use information and increase their problem-solving confidence. In addition, active efforts should be made in nursing university education to increase students’ information utilization ability, such as by organizing nursing curricula and utilizing teaching methods using smart devices and applications. 

### 4.4. Limitations

As the subjects of this study were selected by convenience samples from a specific region, attention should be paid to the generalized interpretation of the study’s results. In addition, it is difficult to explain the causal relationship between these variables in a cross-sectional descriptive study. Further, not encompassing all factors are encompassed, including potential confounders that affect problem-solving confidence. Therefore, for a comprehensive understanding of the subject’s problem-solving confidence, longitudinal studies that confirm causality structurally by considering various characteristics, in addition to the factors presented in this study, as well as repeated studies of a large sample are needed.

## 5. Conclusions

In this study, it was determined that the higher the nursing students’ acceptance intention of a smart device and information literacy, the higher their problem-solving confidence. ICT in the healthcare environment is rapidly changing. Accordingly, to improve nursing students’ problem-solving confidence, educational strategies (such as reinforcement of information science subjects and the use of various educational media) should be supported to enable them to increase their information experience and information utilization capabilities. 

## Figures and Tables

**Table 1:** Scores of acceptance intention of a smart device, information literacy, and problem-solving confidence.

Variables		Acceptance Intention of a Smart Device (Range 1–7)		Information Literacy (Range 1–5)		Problem-Solving Confidence (Range 1–5)	
		Mean (SD)	t (*p*)	Mean (SD)	t (*p*)	Mean (SD)	t (*p*)
Total mean score		4.78 (0.82)		3.87 (0.53)		3.40 (0.37)	
Gender	Male	4.77 (1.02)	−0.118 (0.906)	3.86 (0.55)	−0.144 (0.885)	3.57 (0.33)	3.598 (<0.001)
	(*n* = 56, 12.4%)						
	Female	4.78 (0.79)		3.87 (0.52)		3.38 (0.37)	
	(*n* = 394, 87.6%)						
Grade	Third	4.73 (0.87)	−1.081 (0.280)	3.82 (0.55)	−1.975 (0.048)	3.40 (0.37)	−0.430 (0.667)
	(*n* = 194, 43.1%)						
	Forth	4.81 (0.78)		3.91 (0.50)		3.41 (0.37)	
	(*n* = 256, 56.9%)						

SD: Standard deviation.

**Table 2:** Correlation among variables.

Variables	Acceptance Intention of a Smart Device	Information Literacy	Problem-Solving Confidence
	r (*p*)		
Acceptance intention of a smart device	1		
Information literacy	0.541 (*p* < 0.001)	1	
Problem-solving confidence	0.353 (*p* < 0.001)	0.501 (*p* < 0.001)	1

**Table 3:** Differences in problem-solving confidence according to acceptance intention of a smart device and information literacy (*N* = 450).

Variables	Problem-Solving Confidence	
	Mean (SD)	t (*p*)
Acceptance intention of a smart device		
High score group (*n* = 224, 44.8%)	3.50 (0.36)	5.447 (<0.001)
Low score group (*n* = 226, 45.2%)	3.31 (0.35)	
Information literacy		
High score group (*n* = 228, 45.6%)	3.56 (0.36)	10.072 (<0.001)
Low score group (*n* = 222, 44.4%)	3.24 (0.31)	

Note. SD: Standard deviation.

**Table 4:** Logistic regression of variables according to problem-solving confidence group.

Variables	Problem-Solving Confidence Group ^a^			
	B	*p*	OR ^b^	95% CI
Acceptance intention of a smart device				
Low score group (*n* = 226, 45.2%)			1	
High score group (*n* = 224, 44.8%)	0.728	<0.001	2.071	1.412–3.037
Information literacy				
Low score group (*n* = 222, 44.4%)			1	
High score group (*n* = 228, 45.6%)	1.603	<0.001	4.966	3.298–7.477

Note. OR: Odds Ratio, CI, confidence interval. ^a^ Problem-solving confidence: (1) base group-low score group-232 (51.6%), (2) compare group-high score group-218 (48.4%). ^b^ Adjusted OR by gender, grade.

## Data Availability

Data sharing not applicable: No new data were created or analyzed in this study.
